# Point cloud registration algorithm using liver vascular skeleton feature with computed tomography and ultrasonography image fusion

**DOI:** 10.1007/s11548-025-03496-w

**Published:** 2025-08-23

**Authors:** Satoshi Miura, Masayuki Nakayama, Kexin Xu, Zhang Bo, Ryoko Kuromatsu, Masahito Nakano, Yu Noda, Takumi Kawaguchi

**Affiliations:** 1 Institute of Science Tokyo, Tokyo, Japan; 2 Waseda University, Tokyo, Japan; 3 Kurume University Hospital, Fukuoka, Japan

**Keywords:** Medical robotics, Image fusion, Point cloud registration, Radiofrequency ablation

## Abstract

**Purpose:**

Radiofrequency ablation for liver cancer has advanced rapidly. For accurate ultrasound-guided soft-tissue puncture surgery, it is necessary to fuse intraoperative ultrasound images with preoperative computed tomography images. However, the conventional method is difficult to estimate and fuse images accurately. To address this issue, the present study proposes an algorithm for registering cross-source point clouds based on not surface but the geometric features of the vascular point cloud.

**Methods:**

We developed a fusion system that performs cross-source point cloud registration between ultrasound and computed tomography images, extracting the node, skeleton, and geomatic feature of the vascular point cloud. The system completes the fusion process in an average of 14.5 s after acquiring the vascular point clouds via ultrasound.

**Results:**

The experiments were conducted to fuse liver images by the dummy model and the healthy participants, respectively. The results show the proposed method achieved a registration error within 1.4 mm and decreased the target registration error significantly compared to other methods in a liver dummy model registration experiment. Furthermore, the proposed method achieved the averaged RMSE within 2.23 mm in a human liver vascular skeleton.

**Conclusion:**

The study concluded that because the registration method using vascular feature point cloud could realize the rapid and accurate fusion between ultrasound and computed tomography images, the method is useful to apply the real puncture surgery for radiofrequency ablation for liver. In future work, we will evaluate the proposed method by the patients.

## Introduction

Liver cancer is one of the more common malignant tumors [1]. According to estimates from the International Agency for Research on Cancer, a subsidiary of the World Health Organization, 900,000 people worldwide were estimated to have liver cancer in 2020, with 830,000 expected to die from the disease [2]. Radiofrequency ablation [3] is a rapidly evolving minimally invasive surgery commonly used in the treatment of liver cancer, not only surgical robot [4, 5]. The electrical current kills cells in the tumor around the electrode, inducing the coagulation and necrosis of the tumor tissue.

The ultrasound (US)-guided radiofrequency ablation of liver cancer uses US imaging technology to guide a radiofrequency probe to the liver tumor for radiofrequency ablation [6]. This method combines radiofrequency ablation technology with real-time US imaging to provide more precise positioning and real-time monitoring, allowing doctors to perform surgeries more accurately [7]. Due to the limited image resolution provided by US equipment, it is sometimes impossible to accurately locate the tumor relying only on US images during surgery. Although computed tomography (CT), another commonly used imaging technology, provides higher-resolution images, it cannot be directly used in surgery owing to its slow acquisition of images. To use registration technology in radiofrequency ablation treatment, a CT image obtained before surgery is matched with real-time US imagery recorded during surgery to provide doctors with more information and help them locate a tumor more accurately [8].

The registration step is critical in liver cancer radiofrequency ablation for ensuring that the radiofrequency probe accurately locates the tumor in the liver and performs precise radiofrequency ablation [9]. In the US-guided radiofrequency ablation of hepatocellular carcinoma, alignment refers to matching tumor location information obtained from US imaging to actual patient anatomy (e.g., patient anatomy in CT imaging obtained preoperatively). The alignment problem involves the cross-source medical image matching US and CT images. Real-time matching is difficult using traditional image alignment methods for two-dimensional images owing to their low alignment accuracy and low matching speed [10]. For example, Paccini reported that the automatic fusion 3D US and MR (magnetic resonance)/CT imaging system requires not only US and MR/CT images but also a portable 3D camera and an electromagnetic tracing sensor [11]. Therefore, it is necessary to fuse US and CT images without other sensor tools. To identify and reconstruct liver blood vessels in US and CT images of the liver, the algorithm converts data from two-dimensional space to three-dimensional (3D) space and thus generates a vascular point cloud for both US and CT [12]. After aligning the CT and US point clouds, for any real-time US image, the algorithm can obtain the corresponding CT slice using the coordinate system of the 3D point cloud [13]. In cross-source registration, appreciable differences in shape and density between the two vascular point clouds due to the characteristics of CT and US images and errors in 3D reconstruction make it challenging for traditional or machine learning algorithms to achieve accurate registration results [14].

**Fig. 1:**
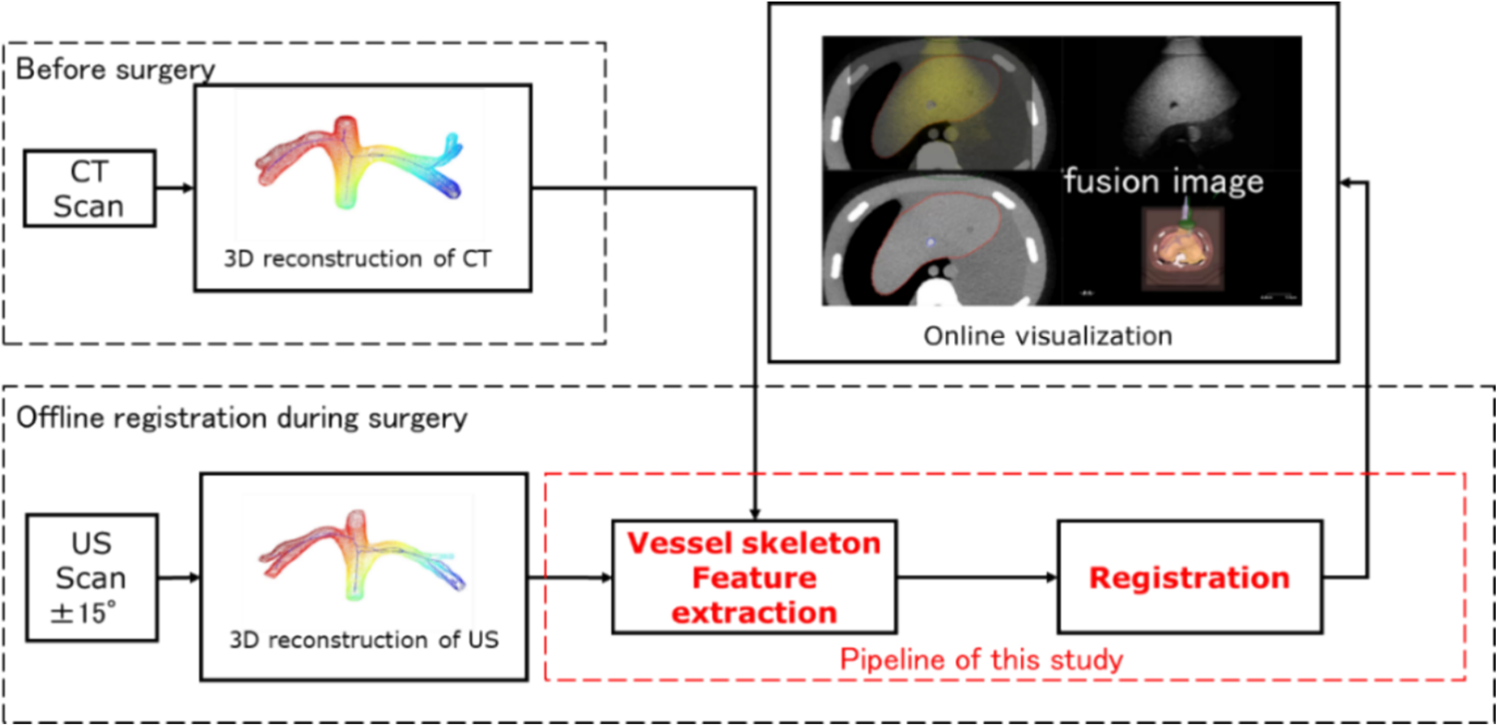
Overview of CT and US image fusion. The flowchart shows the fusion procedure. The red box shows the main issue of this study. Especially, the system estimates not only vascular shape but skeleton of the feature

The aim of this study is to register two types of cross-source point clouds: a vascular point cloud of the liver three-dimensionally reconstructed from US images and vascular point clouds reconstructed from CT images. The goal of registering the cross-source vascular point clouds from CT and US is to fuse CT and US images during radiofrequency ablation treatment, thereby assisting the localization of tumors during treatment. This study proposes a method to extract geometric features of vascular point clouds for registering cross-source vascular point clouds, addressing current challenges in accurately aligning these datasets (Fig. 1). The effectiveness of existing registration algorithms in aligning non-homologous vascular point clouds is limited. Venous blood vessels in the liver have a coronary structure, and topological structures and center lines can be used to simplify the structure of the blood vessels. If the same geometric features (such as center lines) can be extracted from two vascular point clouds, the two vascular point clouds can be simplified from complex structures to simple structures. It is easier to register two vascular point clouds by matching simplified point clouds than by adopting direct registration. The innovation of this study is the conversion of non-homologous vascular point clouds (vascular shape) into homologous point clouds (vascular skeleton feature) that are easy to register by extracting geometric features, which avoids the impact of differences in non-homologous point clouds on registration. While the conventional method uses only the vascular shape, the proposed method uses the vascular skeleton feature. Point clouds based on surface shape are sensitive to missing data, even in lesser amounts, whereas the skeleton feature is more robust, less affected by missing data, and maintains higher accuracy.

## Material and method

### Surgical robot system

**Fig. 2:**
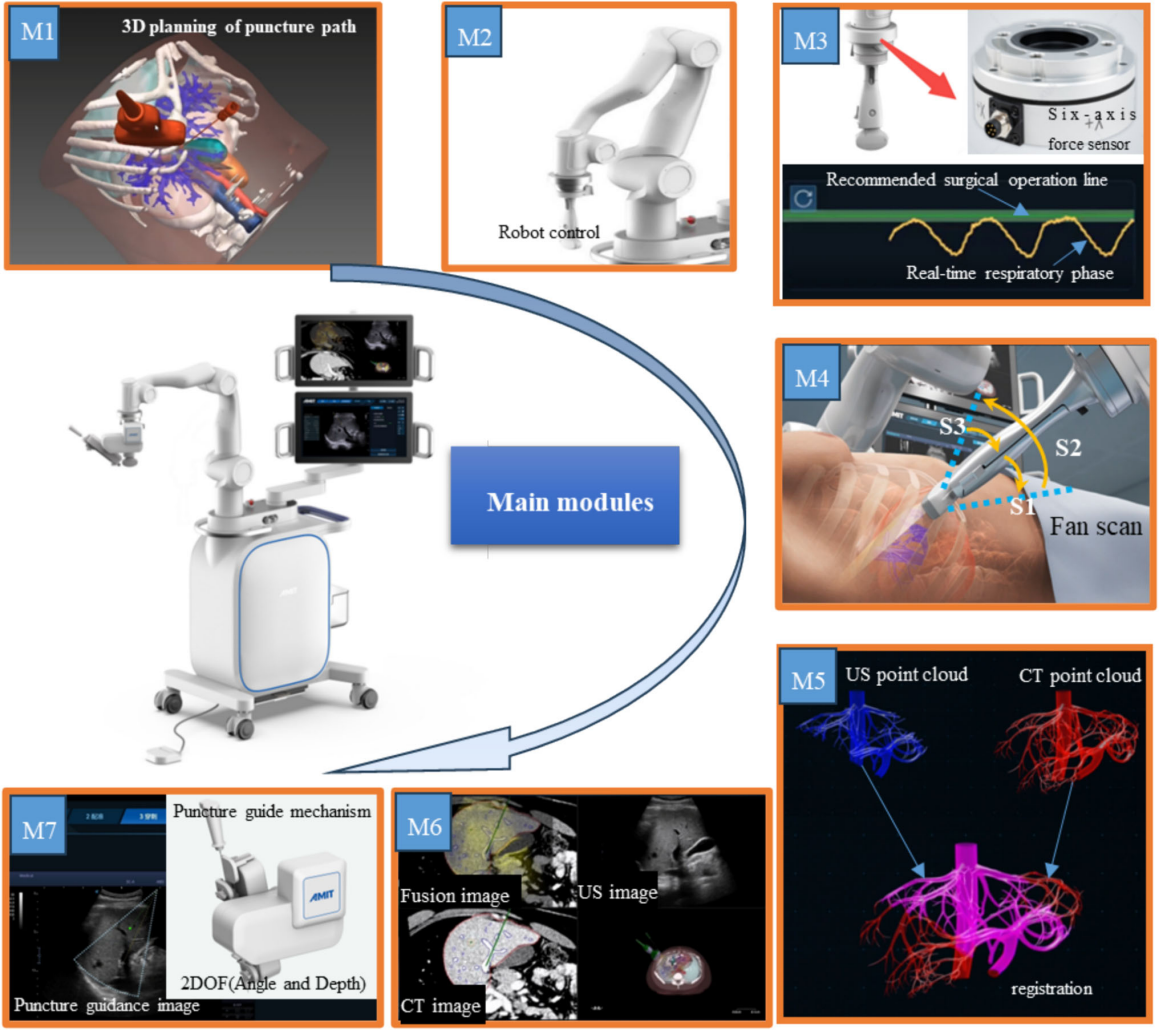
Main modules of the robot. First, the robot plans the puncture surgery before the operation to assist doctors (M1). Second, the doctor can switch between free dragging mode and automatic motion control of the surgical robot (M2). The robot has seven degrees of freedom, and its end is rigidly connected to the US probe. The system can use the positional data of the robotic arm and the information of US images in place of data from the external optical sensors of traditional surgical robots for tumor localization. Third, the force exerted on the ultrasound probe is acquired through a six-axis force sensor integrated with the robot arm (M3). Based on the acquired force data, the respiratory phase of the patient is identified, facilitating the acquisition of US images during the inspiratory phase, and enabling precise navigation of the puncture trajectory. Fourth, the system reconstructs the 3D image using the US probe (M4). The system directs the US probe to perform a fan scan, capturing US information of the liver tissue along the path of the probe. The reconstruction algorithm uses the obtained US images and the position data of the robotic arm to construct a 3D US model. This model is then used to convert real-time US blood vessels into a point cloud. The blood vessel is estimated and converted from the shape to point cloud using the previous AI model [15] which is constructed by the U-Net model and learnt the labeled US image by surgeons. Fifth and sixth, the system registers the image (M5) and fuses 3D US and CT images using this paper’s following method (M6). Finally, the robot performs puncture surgery (M7)

We developed a surgical robot system designed for precise tumor localization, real-time US image fusion, and adaptive puncture guidance. The robot performed measurements following a seven-step procedure, as shown in Fig. 2. All these steps are completed rapidly, taking just a few tens of seconds in total, with the patient only needing to hold their breath briefly. The surgical robot was used for validation of the proposed method.

### Skeleton feature extraction

**Fig. 3:**
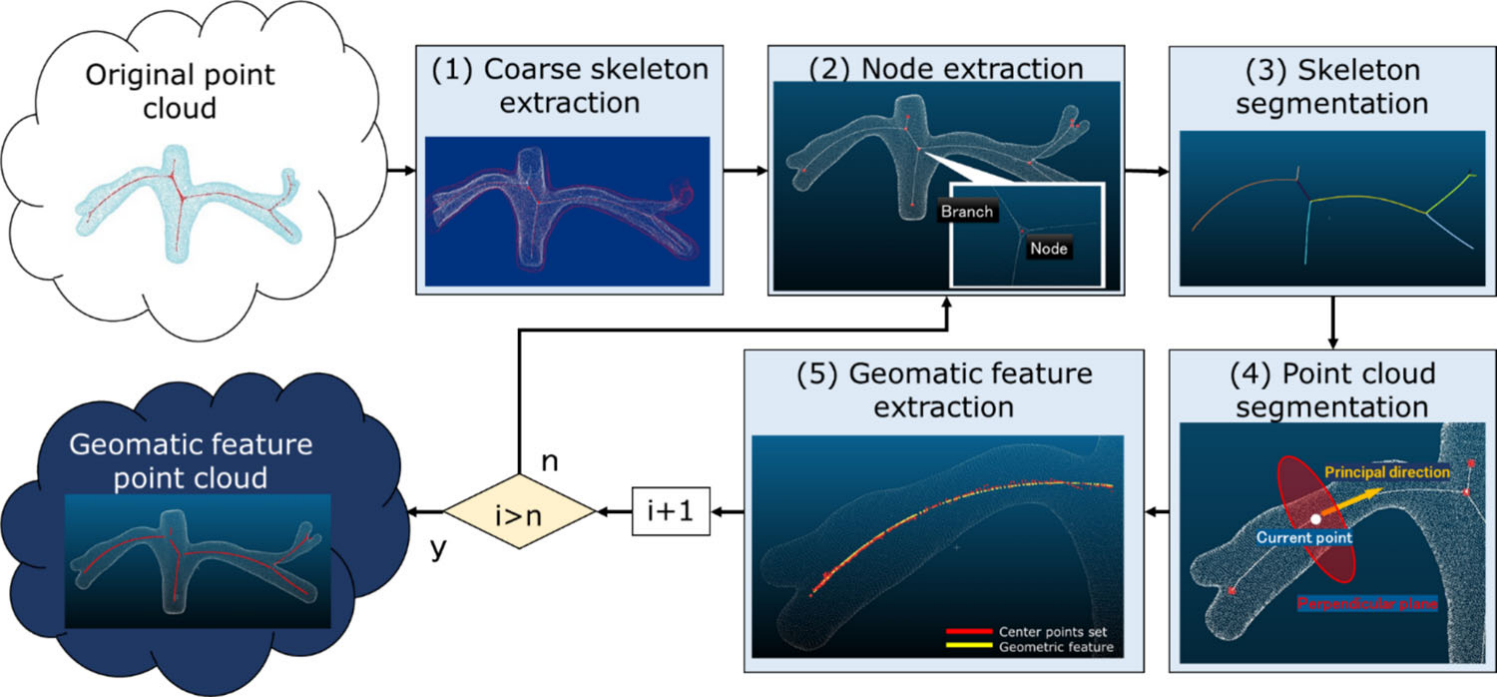
Skeleton Feature Extraction flow. Starting from the original point cloud, the system extracts the coarse skeleton of the blood vessel (**1**) and the nodes (**2**) for construction of the skeleton (**3**). The system uses the point cloud segmentation (**4**) and extracts the geomatic feature extraction (**5**)

We proposed a fusion algorithm that aligns CT and US images using vascular point clouds. The process flow of the point cloud registration algorithm based on geometric feature extraction is described as follows (Fig. 3).

1. Coarse skeleton extraction
  We use a point cloud skeleton extraction algorithm based on the Laplacian operator. The method directly extracts the skeleton without relying on a mesh file. The neighborhood information of the point cloud is used in the Laplace operator-based point cloud shrinkage algorithm to construct the Laplace weighting matrix. In this algorithm, the *k* nearest neighbors are used to build the neighborhood information of the points, making it more suitable for sampling uneven point clouds. The value of *k* determines the number of points in the neighborhood and thus affects the construction of the Laplacian weighting matrix. Therefore, *k* affects the shrinkage of the point cloud to some extent.
  The extracted vascular point cloud skeleton is used only as an intermediate result. Using the extracted vascular point cloud skeleton, we further obtain the features of blood vessels for three reasons. First, as the shape density of the input point cloud varies, it is difficult to use the same set of parameters to adapt to all point clouds for addressing the incomplete contraction or the skeleton flying out of the model. Second, the point cloud skeleton extraction algorithm based on the Laplace operator only roughly extracts the structure of the point cloud. Third, a challenge in skeleton extraction is the lack of a strict definition for the extraction results, which makes it difficult to evaluate their quality.
2. Node extraction
  The point density at the nodes and branches of the shrunken skeleton remains consistent, with generally higher density at the nodes. The coarse skeleton is first traversed using the minimum spanning tree (MST) algorithm to obtain a complete undirected graph containing all the coarse skeleton points. The node has the degree according to the number of the branch (Fig. 4). For example, when the node connects to other three nodes, the node’s degree is three. Next, we optimize the nodes because a simplification of the plural nodes is needed in this situation, as there are several nodes with a degree of three or more in that region of the generated undirected graph. The branch contains nodes with one degree in ends, and the trunk has two nodes in ends with three degrees.

**Fig. 4:**
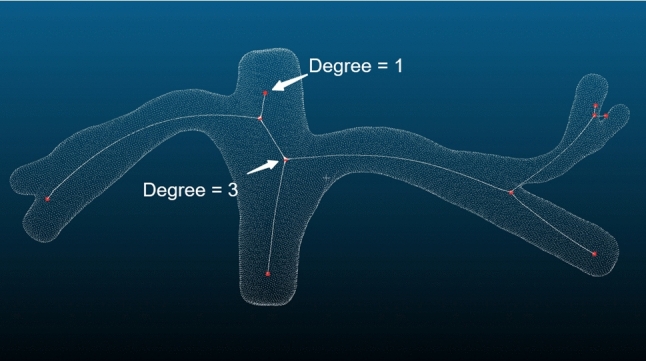
Degree of node. Degree shows how many other nodes can connect
3. Skeleton segmentation
  Using the undirected graph, we obtain the set of node indices that represent the connectivity relations of a node. Next, the coarse skeleton is split depending on the relationships between nodes. A pair of nodes (respectively, referred to as the start node and end node) are selected according to the connections between nodes in the undirected graph for each split. The start node is node with one degree, and the end node is node with three degrees. Using Dijkstra’s algorithm, the shortest path from the start node to the end node is determined within the complete skeleton undirected graph. All the points traversed along this path are recorded, and they are then removed from the coarse skeleton point cloud. After the same operation is performed on all the node connectivity relations, the coarse skeleton point cloud is split into different small segments according to the nodes.
4. Point cloud segmentation.
  The original vascular point cloud is segmented into small segments according to the segmented skeleton, and the geometric center is calculated for each small segment as follows. First, we iterate through all the points in the skeleton segment and calculate the principal direction of the skeleton adopting principal component analysis (PCA) with neighborhood $k = 20$. We sort the skeleton point cloud according to the principal directions. Second, we iterate through the sorted skeleton point cloud, take the skeleton point as the center of the circle, use a *k*-dimensional tree to search for the original vascular points within a radius *r* = 15 mm of each skeleton point, and obtain a set of preliminarily segmented point clouds $[{P}_{1},{P}_{2},\dots \dots ,{P}_{n}]$ because the radius 15 mm is not too wide and narrow to collect the appropriate point cloud.
5. Geometric feature extraction.
  The geometric feature curves are obtained from the segmented point clouds. Each set of preliminary segmented point clouds is calculated as follows. First, the system calculates the center point $${C}_{i}$$ of the point cloud $$ {P}_{i}$$ and the principal axis and vertical direction of the current group. Second, the system calculates the two points $${A}_{i}$$ and $${B}_{i}$$ of the original vascular point cloud closest to the center point $${C}_{i}$$ in the vertical direction and calculates the distance $${d}_{i}$$ between them. Third, the system takes the half distance $${d}_{i}/2$$ as the radius and the center point $${C}_{i}$$ as the center and finds a circle of neighbors $${N}_{i}$$ of the center point $${C}_{i}$$ in the original vascular point cloud. Finally, the system calculates the center point $${C}_{i}{\prime}$$. The system repeats these four tasks to obtain a set of recalculated center points $$[{C}_{1}{\prime},{C}_{2}{\prime},\dots \dots ,{C}_{n}{\prime}]$$ of the original vascular point cloud. Finally, it uses a quadratic polynomial to fit the center points $$\left[{C}_{1}{\prime},{C}_{2}{\prime},\dots \dots ,{C}_{n}{\prime}\right]$$ and obtains the fitted feature curves.

### Registration

**Fig. 5:**
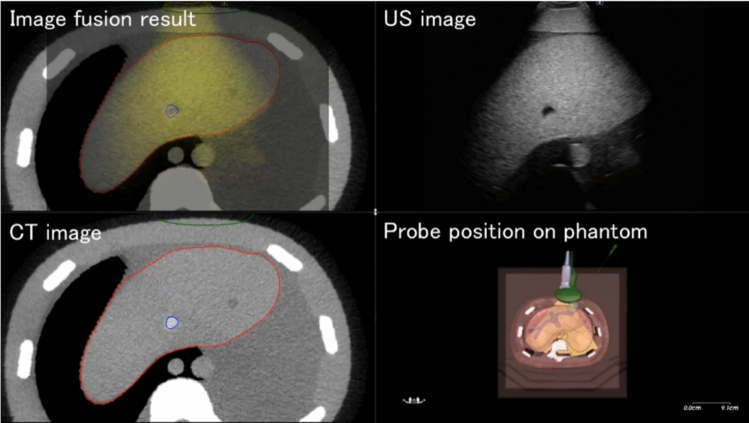
Fusion image between real-time US and CT images. The upper left shows the fusion image. The upper right shows the US image. The lower left is CT image. The lower right is probe position on phantom

Upon completing the above steps, we obtain the geometric features of the vascular point cloud segments. The point clouds from CT and US are extracted separately, allowing us to obtain the geometric features of the CT point cloud and US point cloud individually. Next, the geometric features of the US are taken as the source, and the geometric features of the CT are used as the target in performing the registration operation. The algorithms used for registration are global optimal iterative closest point (GO-ICP) and iterative closest point (ICP). A previous study found that secondary registration using ICP after GO-ICP can improve registration accuracy. In the present study, the two extracted feature point clouds were aligned in two stages. GO-ICP was used for the first coarse registration, and ICP was used for the second fine registration. The parameter settings for GO-ICP and ICP registration were optimized, as presented in Tables 1 and 2, respectively. The US had a refresh rate of 18 fps. The registration results are shown in Fig. 5. After the registration, the system transformed the matrix of the CT image according to the US image. On average, it took us 14.5 s to complete the fusion after obtaining the vascular point clouds by US. If only GO-ICP is used, the fusion time will decrease but the accuracy is also lower. Because the fusion time 14.5 s using the GO-ICP and ICP is enough short to utilize during surgery, we should prioritize the puncture accuracy.

**Table 1:** Parameter settings for GO-ICP registration

Items	Value
rNode.a	− 3.1416
rNode.b	− 3.1416
rNode.c	− 3.1416
rNode.w	6.2832
tNode.x	− 0.5
tNode.y	− 0.5
tNode.z	− 0.5
tNode.w	1.0
Goicp.MSEThresh	0.001
Goicp.trimFraction	0.1

**Table 2:** Parameter settings for ICP registration

Items	Value
nbMaxIterations	20
adjustScale	False
filterOutFarthestPoints	False
randomSamplingLimit	50,000
finalOverlapRatio	100.0/100.0
transformationFilters	0
maxThreadCount	7
minRMSDecrease	10^− 6^

## Validation experiments

### Dummy model

**Fig. 6:**
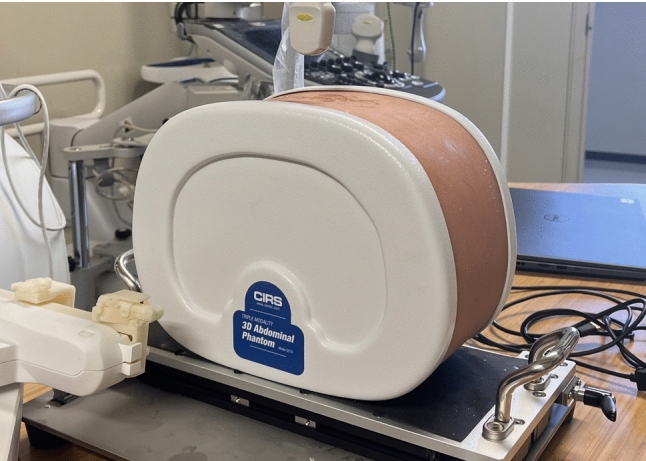
Human abdomen model. The model simulates the 3D abdominal phantom model. The model includes two tumors

**Fig. 7:**
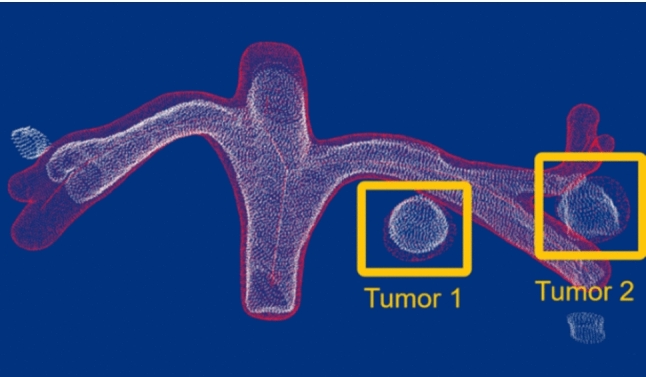
Tumors selected for the evaluation procedure. White point means US-based, and red point means CT-based. The two balls in yellow squared mean tumor

Sixteen pairs of vascular point clouds (hereinafter referred to as body model CT point clouds and body model US point clouds) were obtained through the 3D reconstruction of CT and US images of the liver area in a human model. The human abdomen model (Triple modality 3D Abdominal Phantom Model 057A, CIRS Inc, Virginia, USA) is shown in Fig. 6. In the registration process, the US point cloud was taken as the target, and the CT point cloud was used as the source (i.e., the coordinates of the US point cloud were unchanged), and the CT point cloud was used to align the US point cloud. As the purpose of registration is to better localize a tumor in radiofrequency ablation treatment, the accuracy of the registration near the tumor site is important. In the human model used for this case, four tumors were present in the liver, with all being included in the 3D reconstruction. Different algorithms were evaluated according to the distance between tumor mass centers. Two tumors, shown in Fig. 7, were selected for the evaluation. In addition, to quantitatively evaluate the overall agreement and geometric validity of the segmented regions, three metrics were computed: dimensional and contextual evaluation (Dice), volume similarity (VS), and surface Dice at 5 mm tolerance (SDV5) [16].

### Human body

Eight pairs of vascular point clouds near the portal vein of the liver were obtained through the 3D reconstruction of human CT and US images (hereinafter referred to as the human CT point cloud and human US point cloud). The data were obtained for two healthy men aged 20–30 years. The scans were performed under the supervision of a doctor. To prevent organ displacement due to breathing from affecting image recognition and 3D reconstruction, the participants were asked to hold their breath during the US scans.

Owing to the complexity of the human vascular point cloud, irrelevant branch vessels can interfere with the registration. It is thus necessary to set weights for the geometric features before registration. The extracted geometric features are segmented in this process, with the registration weights applied accordingly. To reduce the effect of the end capillaries on registration, a uniform down-sampling process is applied at the ends of the blood vessels in all feature point clouds, with the down-sampling parameter set at *k* = *5*. We set parameters as listed in Table 3 for the skeleton contraction of vascular point clouds from the body model and human data.

**Table 3:** Skeleton contraction parameters of vascular point clouds

Items	Dummy model	Human
Down sample size	0.8	0.8
Nearest neighbor points *k*	100	100
Initial contraction weight	0.5	0.5
Initial attraction weight	4	10
Max interaction steps	10	10
Termination ratio	0.003	0.003

## Results

### Dummy model

**Fig. 8:**
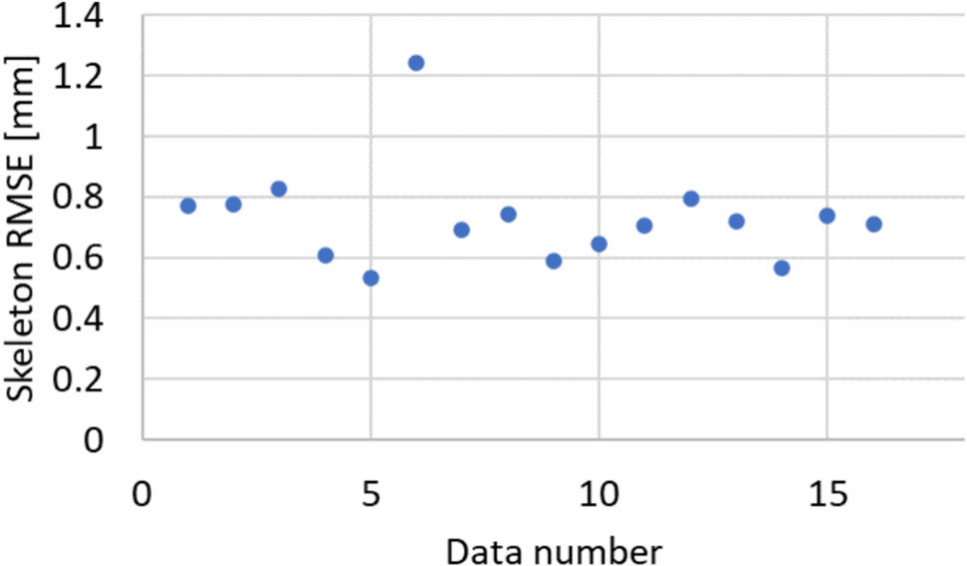
Skeleton RMSE for the body model. The data number means each trial

**Fig. 9:**
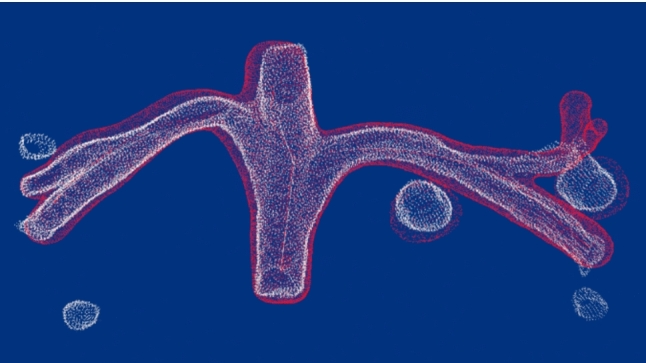
Partial registration results for the body model point cloud. White point shows US-based. Red point shows CT-based

**Fig. 10:**
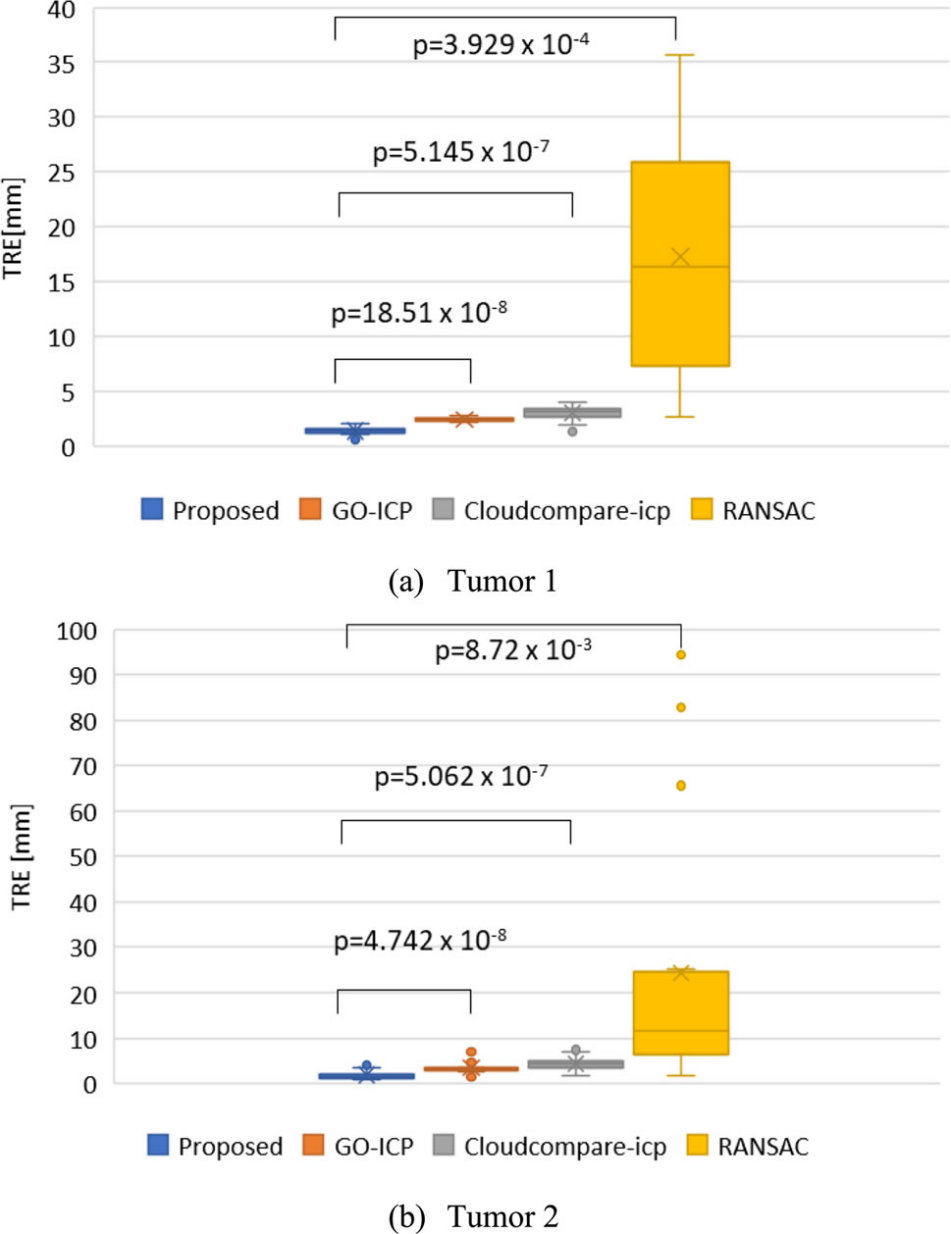
TRE in comparison with algorithms. The bats stamp shows the average. The p-value means probability value

The registration results for the fifteen pairs of mannequin point cloud data are shown in Fig. 8. The average RMSE is 0.728 mm. Figure 9 shows the partial registration results of the human model point cloud data. The target registration error (TRE) of our algorithm is compared with the GO-ICP, the Cloud Compare-ICP, RANSAC in Fig. 10.

The results show that the overall registration error for Tumor 2 is approximately 3 mm larger than that for Tumor 1. This discrepancy is due to the limited scanning range of the US, which may result in the incomplete representation of Tumor 2 in the US point cloud.

Furthermore, the results indicate that our algorithm achieves a smaller average TRE in the registration results of the human model data. In the case of Tumor 1, the average TRE for our algorithm is 1.046 mm smaller than that for GO-ICP and 1.612 mm smaller than that for Cloud Compare-ICP, which are reductions of 43.2% and 53.9%, respectively. In the case of Tumor 2, the average TRE for our algorithm is 1.576 mm smaller than that for GO-ICP and 2.434 mm smaller than that for Cloud Compare-ICP, which are reductions of 44.9% and 55.6%, respectively. Although RANSAC has a minimum value of the TRE similar to the minimum values for the other algorithms, its maximum value is large, showing that RANSAC’s stability in registering cross-source vascular point clouds is poor. A paired t-test reveals a significant difference in the average TRE between our algorithm and the other algorithms (*p* < 0.001). In summary, the results show that our algorithm is more accurate in registration in the region near a tumor.

For Tumor 1, the mean Dice was 0.71, the mean VS was 0.86, and all SDV5 scores were equal to 1. In liver tumor ablation procedures, a 5 mm margin is typically considered to ensure sufficient ablation. Accordingly, all SDV5 scores across all trials indicate the potential clinical utility of the proposed registration method. For Tumor 2, the mean Dice was 0.64 and the mean VS was 0.72, SDV5 scores were lower than 1 in 10 of the 15 cases. The reduction in Dice, VS, and SDV5 scores for Tumor 2 can be attributed to the anatomical location of the tumor near the boundary of the ultrasound scanning range, leading to insufficient spatial inclusion of the lesion.

### Human body

**Fig. 11:**
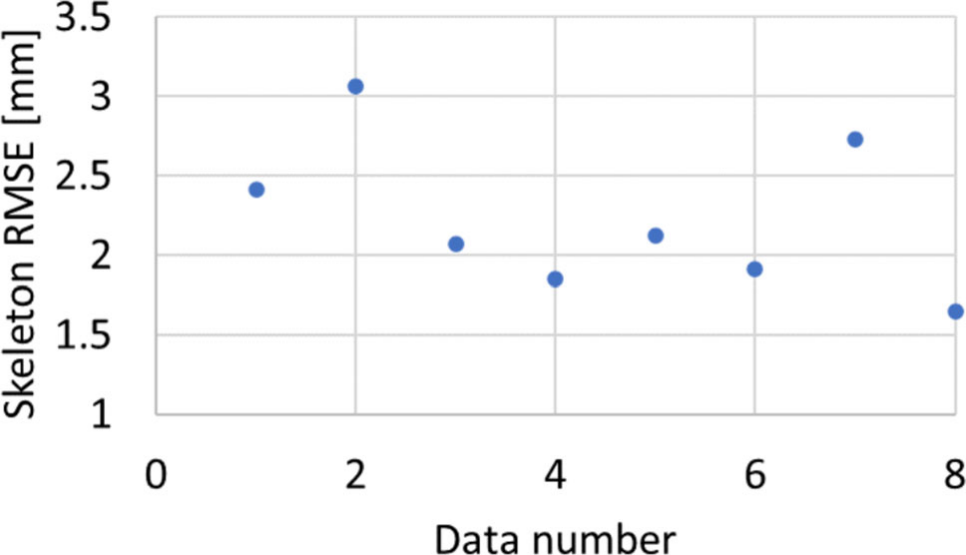
Vascular skeleton RMSE of human data. The data number means each trial

**Fig. 12:**
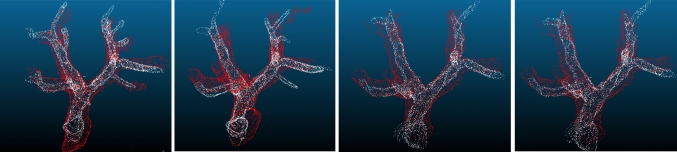
Partial registration results. White point means US-based, and red point means CT-based. The images show the samples among plots in Fig. 11

As the human data were collected from healthy volunteers, tumors are not used for evaluation in the human vascular point cloud data. Instead, the registration results of the different algorithms are evaluated using the RMSE of portal vein vessels point cloud. The average RMSE was 2.23 mm. The registration results for the four pairs of human point cloud data are shown in Figs. 11 and 12.

## Discussion

The TRE results in dummy model indicate the proposed algorithm could significantly improve registration accuracy. Moreover, because the latest related works including deep learning reported the average TRE is higher than 2.5 mm [17, 18], the proposed method is more accurate than the others. Furthermore, the RSME in vascular skeleton feature in both dummy and human model shows the proposed method can estimate the position accurately. Moreover, in the TRE distance index, the distance between the centers of gravity was approximately 1.5 mm. In this system, the basic puncture path is set to aim for the tumor center (center of gravity). Since the error in the TRE is an error in the target puncture position itself, the deviation was shown to be sufficiently smaller than the ablation margin of 5 mm, suggesting its clinical utility.

The Dice value was about 0.71, which was reduced due to various factors. However, our objective is to improve the alignment of the blood vessels, but not to improve the tumor segmentation. In our system, if we make improvements specifically for tumor segmentation, the accuracy of tumor segmentation itself will improve, and we can expect an improvement in Dice score. As can be seen from the Dice values, tumor segmentation accuracy is still incomplete. Even in such a state, the overlap index SDV5 of the surface mesh was 1 in all trials in Tumor1, which had little visible cuts. This meets the margin of ablation surgery of 5 mm, suggesting that even taking into account the imperfections, a reliable ablation is achieved.

Our goal is to achieve the accurate puncture of the tumor utilizing the alignment of the blood vessel. Because the liver inside is like a rigid body, the relative position between tumor and blood vessel does not change during surgery. Hence, if the position of the blood vessel is accurate using the registration, the relative tumor’s position is estimated accurately.

We validated the feasibility by healthy participants in this study. In future work, we will evaluate it by the patients including tumors. Moreover, the proposed algorithm can apply to not only liver surgery but also other surgical applications such as other organ surgery using blood vessels, and alignment of bones. In the alignment of bone, the proposed algorithm can register the point cloud of bones using not the surface but the geometric structural feature of bones. Furthermore, this study’s main issue is the point cloud registration. The initial position alignment is sufficient based on the registration by the study, but we will need to track the estimated pose between tumor and robotic arm in future work.

## Conclusion

This study aligned the 3D point cloud of liver blood vessels reconstructed from US images and the 3D point cloud of non-homologous blood vessels reconstructed from CT images. We proposed a way to extract the geometric features of the vascular point cloud to align the non-homologous vascular point cloud, which addresses a current issue that the non-homologous vascular point cloud cannot be aligned accurately using existing algorithms. In experiments, the average TRE of our proposed algorithm was within 1.4 mm on point cloud data scanned for a human abdomen model. Our algorithm achieved the average RMSE within 2.23 mm on point cloud data scanned for a human body. We thus believe our algorithm has better registration accuracy on the two data types considered in this study.
